# A Fatal Case of Chlorfenapyr Poisoning and the Therapeutic Implications of Serum Chlorfenapyr and Tralopyril Levels

**DOI:** 10.3390/medicina58111630

**Published:** 2022-11-11

**Authors:** Ming-Jin Chung, Yan-Chiao Mao, Chia-Tien Hsu, Mu-Chi Chung, Tsai-Jung Wang, Tung-Min Yu, Po-Yu Liu, Pin-Kuei Fu, Chia-Ming Hsieh

**Affiliations:** 1 Department of Emergency Medicine, Hualien Armed Forces General Hospital, Hualien 971051, Taiwan; 2 Department of Emergency Medicine, Tri-Service General Hospital, Taipei 114202, Taiwan; 3 National Defense Medical Center, School of Medicine, Taipei 114202, Taiwan; 4 Division of Clinical Toxicology, Department of Emergency Medicine, Taichung Veterans General Hospital, Taichung 407219, Taiwan; 5 College of Medicine, National Chung Hsing University, Taichung 40227, Taiwan; 6 Department of Internal Medicine, Division of Nephrology, Taichung Veterans General Hospital, Taichung 407219, Taiwan; 7 School of Medicine, College of Medicine, National Yang Ming Chiao Tung University, Taipei 112304, Taiwan; 8 Department of Critical Care Medicine, Taichung Veterans General Hospital, Taichung 407219, Taiwan; 9 Rong Hsing Research Center for Translational Medicine, National Chung Hsing University, Taichung 40227, Taiwan; 10 Department of Internal Medicine, Division of Infectious Diseases, Taichung Veterans General Hospital, Taichung 407219, Taiwan; 11 Department of Medical Research, Division of Clinical Research, Taichung Veterans General Hospital, Taichung 407219, Taiwan

**Keywords:** chlorfenapyr, tralopyril, fever, seizure, death

## Abstract

Chlorfenapyr is a new contact and stomach insecticide derived from natural pyrroles secreted by *Streptomyces* spp. It is a pro-insecticide and acts after metabolic transformation to its active metabolite tralopyril. Tralopyril is an uncoupler of oxidative phosphorylation in the mitochondria of the target insects and of experiment animals, leading to the disruption of adenosine triphosphate synthesis and death. Several fatal human poisonings had been reported and no blood chlorfenapyr or tralopyril measurements were available. The treatment remains supportive. A 32-year-old healthy man ingested 200 mL of 10% chlorfenapyr as a suicide attempt. Unfortunately, he succumbed at 157 h post-ingestion, shortly after having fever and seizures. His serum level of chlorfenapyr at 4 h post-exposure was 77.4 ng/mL, and was undetectable at 113 and 156 h, respectively. The serum levels of tralopyril were 723.6, 14,179, and 9654.2 ng/mL at 4, 113, and 156 h post-ingestion, respectively. The delay in the rise of serum tralopyril levels was noticeable, which seems to correlate with the patient’s signs and symptoms. The information may have therapeutic implications in the management of this deadly poisoning.

## 1. Introduction

Chlorfenapyr is a relatively new insecticide registered by the United States Environmental Protection Agency in 2001 [1]. Following metabolic transformation to tralopyril, it acts as an uncoupler of mitochondrial oxidative phosphorylation after contact or ingestion by target insects or application to rodent cells [2,3,4]. Fatal human cases with present with a delayed onset of signs and symptoms, including diaphoresis, tachypnea, tachycardia, mental change, fever, and eventually cardiac asystole after chlorfenapyr poisoning suggesting involvement of toxic metabolites, have been reported in 2004 [5]. Typically, there is a latent period of several days between the ingestion and onset of the signs and symptoms, and this period may give a false sense of security to clinicians. Once fever occurs in the later stages, mortality is inevitable [6]. There is no antidote, and the treatment remains supportive. We present a fatal case of chlorfenapyr ingestion in which the serum chlorfenapyr and tralopyril levels were measured. We hope that the study’s findings will be helpful to better understand chlorfenapyr poisoning among first-line medical staff and possibly improve the management of these patients.

## 2. Case Report

**Figure 1:**
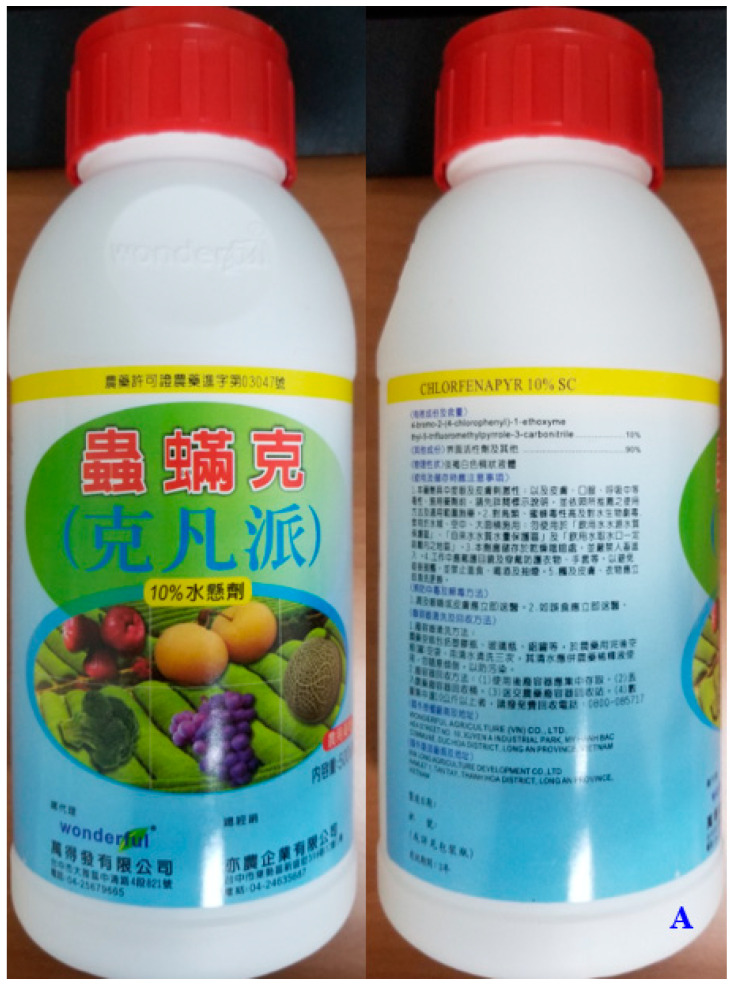

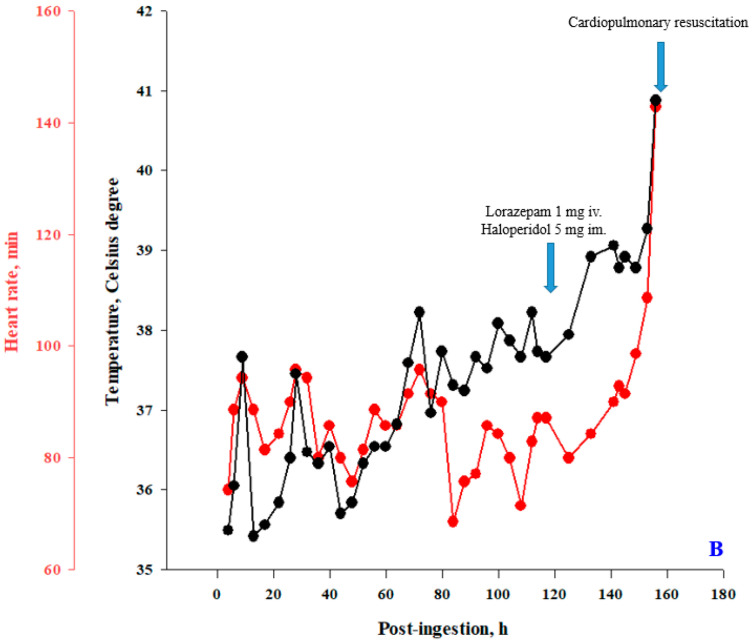
(**A**) The pesticide bottle brought with the patient (anterior and posterior views). (**B**) The patient’s temperature and heart rate versus time post-ingestion. Blue arrows indicate the drugs administered and the start of cardiopulmonary resuscitation.

A 32-year-old, previously healthy, man ingested 200 mL of 10% chlorfenapyr in a suicide attempt because of economic stress. He was sent to a primary care facility by his family with the pesticide bottle (Figure 1A) and received gastric lavage and activated charcoal administration within 1 h of post-ingestion. He was referred to our hospital 4 h post-exposure. On arrival, the patient’s blood pressure (BP) was 133/84 mmHg, pulse 67 beats/min, respiratory rate (RR) was 18 breaths/min, and body temperature was 36 °C. Physical examination revealed modestly epigastric upset. Serial laboratory data are summarized in Table 1. Electrocardiography and chest plain film were unremarkable. He was then admitted to the observation bed.

During hospitalization, he felt hot and perspired at 24 h post-ingestion; however, no fever was observed. At the same time, the urine screen for drug abuse (i.e., amphetamines and morphine) was negative. Subsequently, he progressively developed body weakness and remained in the bed for most of the time. At 114 h post-ingestion, because he had dizziness, a non-contrast-enhanced brain computed tomography was performed but no organic lesions were found. At 122 h post-ingestion, he became delirious with visual hallucinations; therefore, 1 mg lorazepam intravenously and 5 mg haloperidol intramuscularly were administered; however, his delirium worsened, and he was not cooperating. At 153 h post-ingestion, a high fever of up to 38.4 °C and a comatose consciousness (Glasgow coma scale, E1V1M2) were observed. At 156 h post-exposure, a brief tonic seizure followed by cardiac asystole developed and unfortunately, the patient died 157 h (6.5 days) post-ingestion despite immediate cardiopulmonary resuscitation. The vital signs taken 15 min before the cardiac arrest were BP 168/82 mmHg, pulse 144 beats/min, RR 26 breaths/min, and body temperature 40.8 °C (Figure 1B).

We analyzed his serum chlorfenapyr and tralopyril levels with the use of gas- and liquid-chromatography with tandem mass spectrometry, respectively. His serum chlorfenapyr levels at 4 h post-exposure was 77.4 ng/mL and undetectable at 113 and 156 h, respectively; while his serum tralopyril levels were 723.6 ng/mL, 14,179 ng/mL, and 9654.2 ng/mL at 4, 113, and 156 h, respectively. An autopsy was not performed.

## 3. Discussion

Chlorfenapyr was designed from halogenated pyrroles produced by *Streptomyces* spp. and showed enhanced insecticidal activity but diminished mammalian toxicity [1,2,3]. Chlorfenapyr acts as a pro-insecticide that must be converted via oxidative removal of the N-ethoxymethyl group by the microsomal monooxygenase system of target insects to produce the toxic metabolite tralopyril. Tralopyril was reported the most toxic among various metabolites in a rat animal study [7]. The acute toxicity measured by the median lethal dose (LD_50_) following oral ingestion of chlorfenapyr was 441 mg/kg and 45 mg/kg in male rats and mice, respectively [8]; while the oral LD_50_ of tralopyril in male rats was 27 mg/kg [7]. Tralopyril, with both lipophilic and acidic properties, exerts its lethal effect on the insects or rodent cells through mitochondrial uncoupling [2,3,4]. In insect studies, inhibition of microsomal monooxygenase by the specific inhibitor pyperonyl butoxide dramatically reduced the potency of chlorfenapyr, but not tralopyril [2,3]. The detergents in pesticides may enhance gastrointestinal absorption of chlorfenapyr in as reported in an animal study [9].

A total of 13 cases, including two survivors of chlorfenapyr poisoning, have been reported in English literature [6,10,11,12,13,14,15,16,17,18,19,20,21]. Following oral ingestion of chlorfenapyr in humans, the typical presentation is self-limited vomiting and diarrhea, subjective feeling of heat, and diaphoresis on days 1–14 post-exposure [10,11,17,18,20], and restlessness and confusion on days 4–18 [10,11,14,17,18,19,20,22]. High body temperature is usually observed at 5–19 days post-exposure and probably heralds death [10,11,13,14,17,18,20]. The minimal lethal dose appears to be 10 mL (10%; in a 13-year-old girl) through oral administration [20], and the median time to death is 10 days (range: 5–20 days) [6,10,11,13,14,17,18,20,21]. On the other hand, Hoshiko et al. reported a 55-year-old male who sprayed diluted chlorfenapyr (125 mL at 10% in 500 L water) in farming work, and he died on day 7 shortly after developing fever and seizures, possibly related to inhalational and contact exposure [23]. Han et al., reported a 49-year-old male who died 6 days after skin contact with 10% chlorfenapyr solution on his arm, chest, and abdomen [19]. Lee et al., reported a 74-year-old male who died 12 days after self-injection with 20 mL chlorfenapyr into his abdomen [12]. In addition, subjects may be misdiagnosed as acute disseminated encephalomyelitis, which is unresponsive to standard treatments if the exposure history is unknown [11,13]. Permanent paraplegia might occur even when a minimal amount is ingested [16].

Little is known about the mammalian pharmacokinetics of chlorfenapyr and even less is known about tralopyril [7,18]. Although our patient succumbed to poisoning, his serum was collected for toxicants analysis. Chlorfenapyr was measured by gas-chromatography tandem mass spectrometry and tralopyril was measured by liquid-chromatography tandem mass spectrometry as described [24,25,26,27] with modifications. A low level of serum chlorfenapyr was observed 4 h post-exposure, and it was further undetectable on days 4.7–6.5 post-exposure despite significant ingestion of the chlorfenapyr-containing insecticide. A late upstroke of serum tralopyril levels was observed, which possibly more accurately correlates with the patient’s symptoms than that of serum chlorfenapyr. Jina Lee et al., suggested early hemodialysis to treat the poisoning [12]; whereas James Chomin et al., suggested late hemodialysis was futile [18]. Our findings may be helpful to better determine the timing and modality of extracorporeal elimination following chlorfenapyr poisoning.

The major limitation of this study was that the other metabolites were not measured, which have been detected in various animals studied following chlorfenapyr exposure [8]; and hence, their contributions to the poisoning could not be characterized [7]. Moreover, only three serum toxicant levels were obtained; therefore, the exact time that toxicants peaked in human circulation following oral ingestion remained undetermined.

## 4. Conclusions

Following deliberate ingestion of chlorfenapyr-containing insecticide, there is a latent period of several days between the ingestion and onset of signs and symptoms, and this period may give a false sense of security to clinicians. Vigilant monitoring of patients’ serum toxicant levels may be helpful to better understand and management of poisoning cases. The delayed increase in the metabolite levels may more accurately correlate with patient’s clinical manifestations compared with the parent compound levels.

## Figures and Tables

**Table 1:** Laboratory findings of the poisoned case.

Post-Ingestion, h	4	113	156
White blood cell count, 3.9 × 10^9^/L–10.6 × 10^9^/L	9.6	4.97	8.39
Differential count (%)			
Neutrophil	84.9	63.6	89.5
Lymphocyte	9.6	27.4	--
Monocyte	4.6	7.2	--
Hemoglobin, 12.3–18.3 g/dL	14.7	15.3	14.5
Platelet count, 150 × 10^9^/L–400 × 10^9^/L	201	232	--
Sodium, mEq/L	145	138	142
Potassium, mEq/L	3.9	3.7	4.1
Calcium, 8.5–10.1 mg/dL	--	8.7	--
Chloride, mEq/L	115	--	--
Blood urea nitrogen, 5–25 mg/dL	12	28	--
Creatinine, 0.7–1.4 mg/dL	0.7	1	1.1
Glucose, mg/dL	89	159	--
Alanine transaminase, 10–50 U/L	19	63	--
Alkaline phosphatase, 50–190 U/L	--	94	--
Total bilirubin, 0–1 mg/dL	--	0.58	--
Ammonia, 25–94 μg/dL	--	47	--
Prothrombin time, 9.8–14.2 s	--	11.9	--
Amylase, 20–140 U/L	40	--	--
C-reactive protein, <0.3 mg/dL	--	0.08	0.15
Creatine kinase, 10–160 U/L	--	5843.4	8029.8
Troponin-I, <0.2 ng/ml	--	0.02	0.03
Osmolarity, 275–295 mOsm/Kg	286	--	--
Lactate, 3–12 mg/dL	9.4	--	--
Blood gas analysis	(Artery)	(Vein)	(Vein)
pH	7.38	7.44	7.36
O_2_ (mmHg)	103	47.4	21.7
CO_2_ (mmHg)	40.3	44.3	46
Bicarbonate (mmol/L)	23.3	29.1	25.4
Toxicological analysis, (serum; ng/mL) *			
Chlorfenapyr	77.4	UQ	UQ
Tralopyril	723.6	14,179	9654.2

* Limit of quantification was 25 ng/mL for both chlorfenapyr and tralopyril. --: no data available; UQ: unquantifiable (i.e., <25 ng/mL).

## Data Availability

Not applicable.
